# Radiosynthesis and Quality Control of [^67^Ga]-3,4-dimethoxylated Porphyrin Complex as a Possible Imaging agent 

**Published:** 2013

**Authors:** Azadeh Paknafas, Yousef Fazaeli, Amir Reza Jalilian, Abbas Ahmadi, Shahzad Feizi, Mohsen Kamalidehghan, Ali Rahiminejad, Ali Khalaj

**Affiliations:** a *School of Radiation Applications, Nuclear Science and Technology Research Institute (NSTRI), Tehran, Iran, P.O.Box: 31485-498. *; b *Department of Chemistry. Karaj Branch, Islamic Azad University, Karaj, Iran. *; c *Department of Medicinal Chemistry, Faculty of Pharmacy, Tehran University of Medical Sciences, Tehran, Iran. *

**Keywords:** Dimethoxy porphyrins, Ga-67, Biodistribution, Imaging

## Abstract

Radiolabeled porphyrins are potential tumor avid radiopharmaceuticals because of their impersonation in the human body, ability to complex various radionuclides, water solubility, low toxicity *etc*. In this work a radiogallium porphyrin complex has been developed. [^67^Ga] labeled 5,10,15,20-tetrakis(3,4-dimethoxyphenyl) porphyrin ([^67^Ga]-TDMPP) was prepared using freshly prepared [^67^Ga]GaCl_3_ and 5,10,15,20-tetrakis(3,4-dimethoxyphenyl) porphyrin (H_2_TDMPP) for 60 min at 100°C. Stability of the complex was checked in final formulation and human serum for 24 h, followed by biodistribution and imaging studies in wild type rats up to 24 h. The radiocomplex was obtained with radiochemical purity >99% (ITLC) and >98% (HPLC), specific activity: 12-15 GBq/mmol. The partition coefficient was determined (log P=1.63). A detailed comparative pharmacokinetic study performed for ^67^Ga cation and [^67^Ga]-TDMPP. The complex was mostly washed out from the circulation through kidneys. Myocardial uptake was significantly observed by SPECT and biodistribution studies. Knee and shoulder joints demonstrated significant activity uptake in 2h post injection**. **Higher water solubility of the complex due to ionic nature of the complex is an advantage for rapid wash-out of the complex from the system, the complex has significant joint uptake compared to other radiolabeled porphyrins which the mechanisms are explained.

## Introduction

Porphyrins appeal large attention because of their impersonation in the human body, ability to accumulate in many kinds of cancer cells, as well as magnetic and optical properties. Photofrin has currently been approved for general use by licensing authorities for treatment of solid tumor and cancer using photodynamic therapy (PDT) that is based on photochemical effect induced by light (1). Recently, meso-tetra (3-hydroxyphenyl) porphyrin has been developed as one of best tumor localizer and also shown a favorable tissue distribution. These features make them useful in cancer medicine and photodynamic therapy (2, 3). Also tumor accumulation of some gallium- porphyrin complexes has already been reported (4). Radiolabeled porphyrins have been developed for the therapeutic purposes such as, ^109^Pd-protoporphyrins (5), ^109^Pd-porphyrins (6), ^109^Pd-derivitized porphyrins (7) and ^188^Re-porphyrins (8). 

Some diagnostic radiolabeled porphyrins have also been reported. For instance, ^99m^Tc-porphyrin conjugate been evaluated in rodent mammary tumors (9). Recently ^99m^Tc-porphine has been developed for imaging despite the high hepatotoxicity (10). On the other hand the kinetic studies for ^111^In incorporation into *m*-tetraphenylporphine have been studied while no data was reported for its biological evaluation (11).

The interesting physical properties of gallium-67 including physical half life (T_1/2_: 78 h), allowing to design time-consuming preparation and purification steps, gamma photopeaks (185 and 300 keV), suitable for SPECT imaging using most available gamma cameras and SPECT systems, suitable mode of decay (electron capture to 67Zn), a stable daughter, leading to no new radiation peaks and overexposure to patients and also availability in the country make it an interesting nuclide for radiopharmaceutical research (12). 

The increasing trend in the production and use of PET radionuclides in nuclear medicine has offered new opportunities for researchers to focus on the production of new Ga-radiopharmaceuticals for feasibility studies using Ga-67 for their future PET gallium homologs. 

In continuation of our research projects for development of radiopharmaceuticals for human use in the country (13, 14), we are interested in developing new targets at the cellular level.

**Figure 1:**
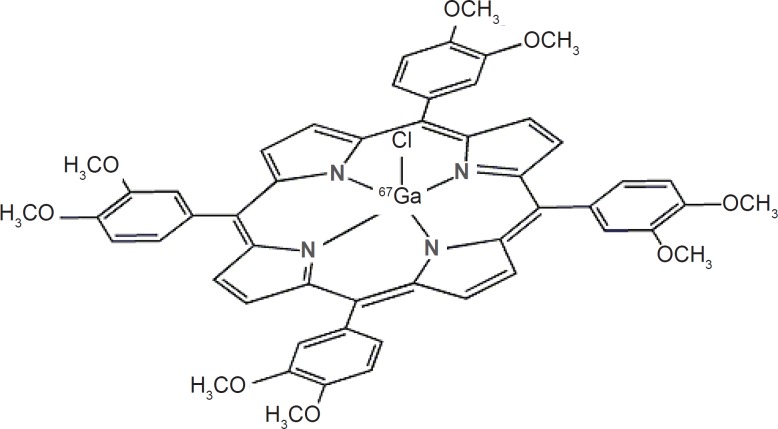
Structure of ^67^Ga-TDMPP

Recently various radiogallium labeled-porphyrin complexes have been reported in the literature (15, 16). Due to the interesting pharmacological properties of porphyrins such as solubility in serum, rapid wash-out, tumor avidity and feasible complexation with various bi/tri-valent metals (17), the idea of developing a possible tumor imaging agent using SPECT (photon emission computed tomography) by incorporating ^67^Ga into a suitable porphyrin ligand, *i.e*. H_2_TFPP was investigated (Figure 1). Production and evaluation of ^67^Ga-TDMPP for diagnostic purposes can lead to the development ultimate Ga-68 homolog compound for PET applications.

In this work we report, synthesis, radiolabeling, quality control, stability, partition coefficient determination and biodistribution studies (using SPECT and scarification) of ^67^Ga-TDMPP in wild-type rats has been described. The time/activity diagrams for the labeled compound in vital organs have been plotted compared to gallium cation.

## Experimental

Enriched zinc-68 chloride with a purity of more than 95% was obtained from Ion Beam Separation Group at Agricultural, Medical and Industrial Research School (AMIRS). Production of ^67^Ga was performed at the Nuclear Medicine Research Group (AMIRS) 30 MeV cyclotron (Cyclone-30, IBA). Other chemicals were purchased from the Aldrich Chemical Co. (Germany); and the ion-exchange resins from Bio-Rad Laboratories (Canada). NMR spectra were obtained on a FT-80 Varian instrument (80 MHz) with tetramethylsilane as the internal standard. Thin layer chromatography (TLC) for cold compounds was performed on polymer-backed silica gel (F 1500/LS 254, 20 9 20 cm, TLC Ready Foil, Schleicher & Schuell, Germany). Normal saline and sodium acetate used for labeling were of high purity and had been filtered through 0.22 mL Cativex filters. Instant thin layer chromatography (ITLC) was performed by counting Whatman No. 2 papers using a thin layer chromatography scanner, Bioscan AR2000, Bioscan Europe Ltd. (France). Analytical high performance liquid chromatography (HPLC) used to determine the specific activity, was performed by a Shimadzu LC-10AT, armed with two detector systems, flow scintillation analyzer (Packard-150 TR) and UV–visible (Shimadzu) using Whatman Partisphere C-18 column 250x9x4.6 mm, Whatman, NJ (USA). Analytical HPLC was used to determine the specific radioactivity of the labeled compound. A standard curve was generated to calculate the mass of the final solution. Biodistribution data were acquired by counting normal saline washed tissues after weighting on a CanberraTM high purity germanium (HPGe) detector (model GC1020-7500SL). Radionuclidic purity was checked with the same detector. For activity measurement of the samples a CRC Capintech Radiometer (NJ, USA) was used. All calculations and tissue countings were based on the 184 keV peak. Animal studies were performed in accordance with the United Kingdom Biological Council’s Guidelines on the Use of Living Animals in Scientific Investigations, 2nd ed. 

*Production of* ^6^ *7* *Ga*

^68^Zn(p,2n)^67^Ga was used as the best nuclear reaction for the production of ^67^Ga. Other impurities could be removed in the radiochemical separation process. After the target bombardment process, chemical separation was carried out in no-carrier-added form. The irradiated target was dissolved in 10 M HCl (15 mL) and the solution was passed through a cation exchange resin (AG 50W, H+ form, mesh 200–400, h:10 cm, Ø:1.3 cm) which had been preconditioned by passing 25 mL of 9 M HCl. The column was then washed by 25 mL of 9 M HCl at a rate of 1 mL/min to remove copper and zinc ions. To the eluent 30 mL water and about 100 mL of a 6 M HCl solution was added. The latter solution was loaded on another exchange resin (AG1X8 Cl- form, 100–200 mesh, h: 25 cm, Ø:1.7 cm) pretreated with 6 M HCl (100 mL). Finally, the ^67^Ga was eluted as [^67^Ga]GaCl_3_ using 2 M HCl (50 mL); the whole process took about 60 min. 

*Quality control of the product*

*Radionuclidic purity*

Gamma spectroscopy of the final sample was carry out through in an HPGe detector coupled to a CanberraTM multi-channel analyzer for 1000 sec. 

*Chemical purity control*

This step was carried out to ensure that the amounts of zinc and copper ions resulting from the target material and backing in the final product are acceptable regarding internationally accepted limits. Chemical purity was checked by differential-pulsed anodic stripping polarography. The detection limit of our system was 0.1 ppm for both zinc and copper ions. 

*Preparation of 5,10,15,20-tetrakis(3,4- dimethoxyphenyl) porphyrin (H*_2_ *TDMPP)*

This compound was prepared according to the reported method using freshly distilled 3,4-dimethoxy benzaldehyde, pyrrole and propionic acid followed by oxidation (18). UV (CH_2_Cl_2_) λ_max_ (ε) = 424,520,556,594,650 nm. 1HNMR: -2.7 (NH), 8.93 (H-pryrole),7.80,7.79,7.78 (H_o1_,H_o2_), 7.29, 7.28(Hm), 4.20, 3.97(H_por_Ho_OMe_). 

*Preparation of [* *67* *Ga]-TDMPP*

The acidic solution (2 mL) of [^67^Ga]GaCl_3_ (111 MBq, 3 mCi) was transferred to a 3 mL-borosilicate vial and heated to dryness using a flow of N_2_ gas at 50-60°C. Fifty micreolitres of TDMPP in absolute ethanol (5 mg/mL ≈409 nmoles) was added to the gallium-containing vial followed by the addition of acetate buffer pH 5.5 (450 microliteres). The mixture refluxed at 100°C for 60 min. The active solution was checked for radiochemical purity by ITLC and HPLC. The final solution was then passed through a 0.22 μm filter and pH was adjusted to 5.5-7. 

*Quality control of [* ^67^ *Ga]-TDMPP*

*Radio thin layer chromatography*

A 5 μL sample of the final fraction was spotted on a chromatography whatman No. 2 paper, and developed in mobile phase mixture, 10% NH_4_OAc and methanol 1:1. 

*High performance liquid chromatography*

HPLC was performed at a flow rate of 1 mL/ min, pressure: 130 kgF/cm^2^ for 20 min. HPLC was performed on the final preparation using a mixture of water:acetonitrile 3:2(v/v) as the eluent by means of reversed phase column Whatman Partisphere C_18_ 4.6 × 250 mm. 

*Determination of partition coefficient*

Partition coefficient (log *P*) of [^67^Ga]-TDMPP was calculated by the determination of *P *(*P*= the ratio of specific activities of the organic and aqueous phases). A mixture of 1 mL of 1-octanol and 1 mL of isotonic acetate-buffered saline (pH=7) containing approximately 3.7 MBq of the radiolabeled gallium complex at 37°C was vortexed 1 min and left 5 min. Following centrifugation at >1200 *g *for 5 min, the octanol and aqueous phases were sampled and counted in an automatic well-type counter. A 500 μL sample of the octanol phase from this experiment was shaken again two to three times with fresh buffer samples. The reported log p*-*values are the average of the second and third extractions from three to four independent measurements. 

*Stability tests*

The stability of the complex was checked according to the conventional ITLC method (19). A sample of [^67^Ga]-TDMPP (37 MBq) was kept at room temperature for 2 days while being checked by ITLC at diffrerent time intervals in order to check stability in final product using above chromatography system. For serum stability studies, to 36.1 MBq (976 μCi) of [^67^Ga]- TDMPP was added 500 μL of freshly collected human serum and the resulting mixture was incubated at 37°C for 5 h, Aliquots (5 L) were analyzed by ITLC. 

*Biodistribution in wild-type rats*

The distribution of the radiolabelled complex among tissues was determined for wild-type rats immediately after imaging. The total amount of radioactivity injected into each animal was measured by counting the 1 mL syringe before and after injection in a dose calibrator with fixed geometry. The animals were sacrificed using the animal care protocols at selected times after injection (2 to 24 h), the tissues (blood, heart, lung, brain, intestine, faeces, skin, stomach, kidneys, liver, muscle and bone) were weighed and rinsed with normal saline and their specific activities were determined with a HPGe detector equipped with a sample holder device as percent of injected dose per gram of tissues. Blood samples were rapidly taken from rodent aorta after scarification. 

*Imaging of [* ^67^ *Ga]-TDMPP in wild-type rats*

Images were taken 2, 4 and 24 h after administration of the radiopharmaceutical by a dual-head SPECT system. The mouse-to-high energy septa distance was 12 cm. Images were taken from both normal and tumor bearing mice. The useful field of view (UFOV) was 540 mm×400 mm. 

## Results and Discussion

The existing Ga-67 citrate compound is not a specific tracer due to cross diagnosis in inflammation, infection and malignancies, although is almost non-expensive, it is not a specific tumor imaging compound, on the other hand, the other Ga-68 tracers such as SSTR_2_ antagonists including ^68^Ga-DOTATOC and ^68^Ga-DOTANOC are neither non-expensive nor available at the moment in many parts of the world while covering the diagnosis of some limited number of neuroendocrinal malignancies. The search for developing new radiotracers targeting alternative biological processes is of great interest. 

*Production and quality control of 67Ga*

Gallium-67, in form of GaCl_3_, was prepared by 24 MeV proton bombardment of the 68Zn target at Cyclone-30 on a regular basis. The target was bombarded with a current intensity of 170 μA and a charge of 1400 μAh. The chemical separation process was based on a no-carrier-added method. 

Radiochemical separation was performed by a two-step ion exchange chromatography method with a yield of higher than 95%. Quality control of the product was performed in two steps. Radionuclidic control showed the presence of 93 (40%), 184 (24%), 296 (22%), 378 (7%) keV gamma energies, all originating from 67Ga and showed a radionuclidic purity higher than 99% (E.O.S.). The concentrations of zinc (from target material) and copper (from target support) were determined using polarography and shown to be below the internationally accepted levels, i.e. 0.1 ppm for Zn and Cu (20, 21). 

*Radiolabeling*

Because of the engagement of NH polar functional groups in its structure, labeling of H_2_TFPP with gallium cation affects its chromatographic properties and the final complex is more lipophilic. 

**Figure 2:**
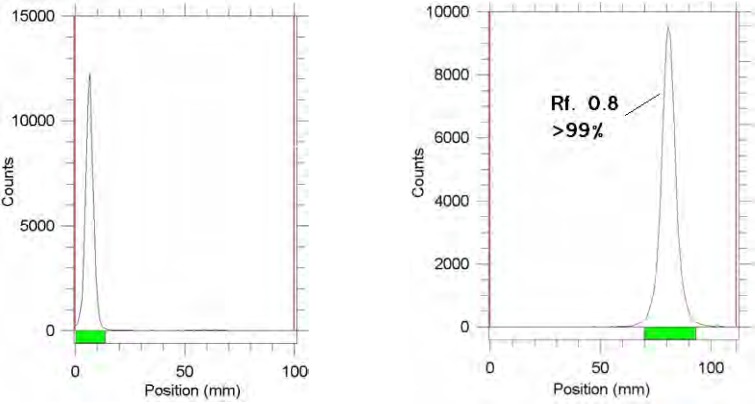
ITLC of [^67^Ga]GaCl_3_ (left) and [^67^Ga]-TDMPP (right) in a 10% NH_4_OAc and methanol 1:1 mixture (left) as mobile phase on Whatman No.2 papers

Chromatographic system was used for the detection of the radiolabeled compound from the free gallium cation. Using 10% NH_4_OAc and methanol 1:1 mixture, free gallium remains at the origin of the paper as a single peak, while the radiolabeled compound migrates to higher Rf (0.8) (Figure 2).

**Figure 3:**
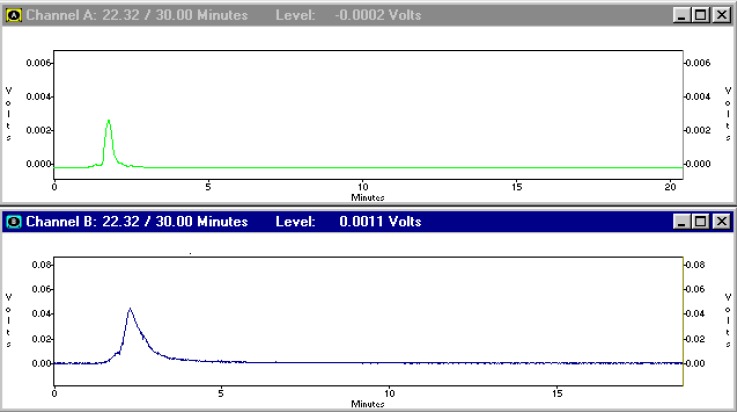
HPLC chromatograms of [^67^Ga]-TDMPP on a reversed phase column using acetonitrile:water 40:60, up; UV chromatogram, down; scintillation chromatogram

Although the ITLC studies approved the production of radiolabeled compound, HPLC studies demonstrated the existence of radiolabeled species using both UV and scintillation detectors. A more fast-eluting compound at 2.27 min (scintillation detector) related to 1.8 min peak (UV detector) demonstrated a more hydrophilic compound. Free Ga cation eluted at 1.02 min (not shown) (Figure 3).

*Partition coefficient of [67Ga]-TDMPP*

As expected from the chemical formula in Figure 1, the lipophilicity of the [^67^Ga]-TDMPP compound is significant due to methoxy groups of the radiocomplex. The measured octanol/water partition coefficient, *P*, for the complex was found to depend on the pH of the solution. At the pH.7 the log P was 1.63. The water solubility of the radiocomplex leads to less unnecessary uptakes in tissues including liver and fat and faster kidney wash-out.

*Stability*

The chemical stability of [^67^Ga]-TDMPP was high enough to perform further studies. Incubation of [^67^Ga]-TDMPP in freshly prepared human serum for 24 h at 37°C showed no loss of ^67^Ga from the complex. The radiochemical purity of complex remained at 98% for24 hunder physiologic conditions. 

*Biodistribution*

**Figure 4:**
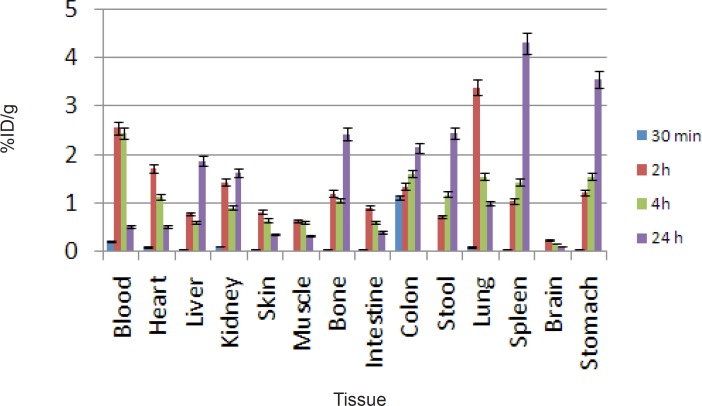
Biodistribution of [^67^Ga]GaCl_3_ (1.85 MBq, 50 μCi) in wild-type rats 0.5-24 h after iv injection via tail vein (ID/g%: percentage of injected dose per gram of tissue calculated based on the area under curve of 184 keV peak in gamma spectrum) (n=5).

For better comparison biodistribution study was performed for free Ga^3+^ as well. The %ID/g data are summarized in Figure 4. As reported previously, ^67^Ga is excreted majorly from gastrointestinal tract (GIT), thus colon and stool activity content are significant while blood stream activity is high at 2–4 h followed by reduction in 24. Bone uptake is also observed after 24 h post injection.

**Figure 5:**
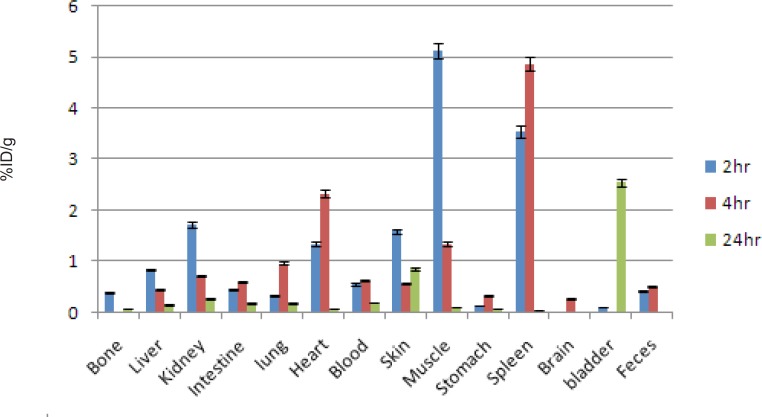
Biodistribution of [67Ga]-TDMPP (1.85 MBq, 50 μCi) in wild type rats 2,4 and 24 h after iv injection via tail vein (ID/g%:percentage of injected dose per gram of tissue calculated based on the area under curve of 184 keV peak in gamma spectrum) (n = 3).

The radiolabeled compound biodistribution is also demonstrated in Figure 5. Due to water solubility of porphyrin compounds the major activity in 2 h post injection is present in the kidneys followed by major accumulation in bladder 24 h post injection, thus the major route of excretion for the labeled compound is urinary tract likewise other similar complexes reported (14, 15). 

Interestingly a significant accumulation of the complex was observed in striated muscles as well as myocardium especially 2 h post injection in thigh muscle and after 4 h in the myocardium. 

Although no direct radioactive accumulation has been reported for radiolabeled porphyrins, it has been shown that myoglobin heme iron could potentially serve as a Fenton reagent for the intracellular generation of hydroxyl radicals, which are responsible for the oxidation of the porphyrins and cellular accumulation (22). Also other reports demonstrate the pharmacological activity of some porphyrins among skeletal muscle via ion channel activation (23). 

**Figure 6:**
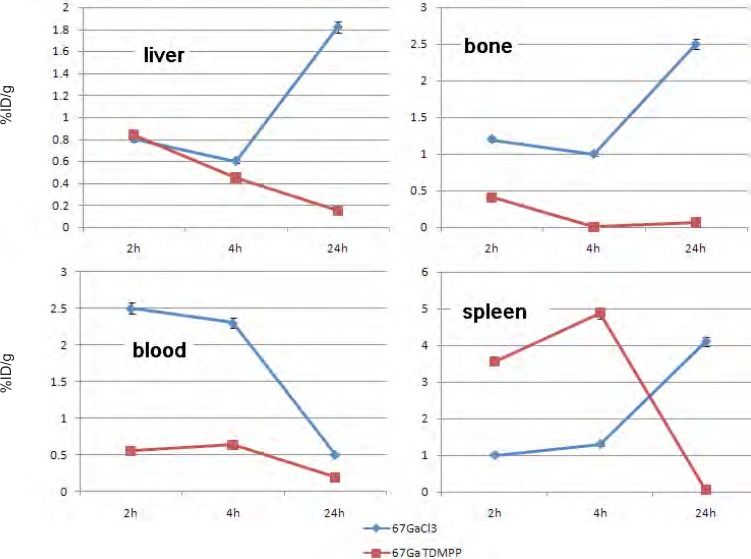
Comparative liver, bone, blood and spleen activity for ^67^Ga-TDMPP and ^67^GaCl_3_ in wild-type rats.

Comparison of vital organs uptake for 67Ga- TDMPP and 67GaCl3 demonstrates kinetic pattern difference for both species (Figure 6.). ^67^Ga cation is accumulated in the liver in the first 24 h post injection slightly, while ^67^Ga-TDMPP second major excretion route is through the liver and the uptake is diminished through the time. 

As shown earlier, ^67^Ga cation is slightly absorbed in the skeletal system (2.5-3%) while the labeled compound almost shows no uptake in the bone in 24 h. 

Both species are present in the circulation in 2 h, Ga-67 is majorly transported by the metaloproteins into liver while the complex is rapidly washed out through the excretion routs. Since the gallium cation can be trapped in blood cells due to the high content of thio-proteins, the spleen activity content is drastically higher in this organ after 24 h compared to the radiolabeled compound. 

*Imaging of [67Ga]-TDMPP in wild type rats*

**Figure 7:**
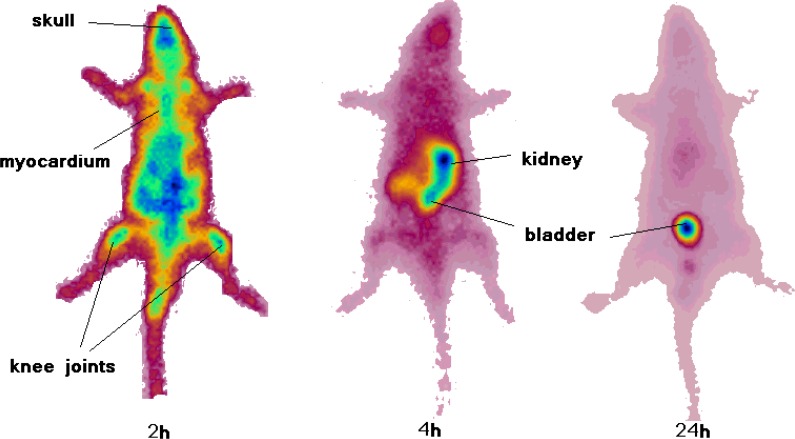
SPECT images of ^67^Ga-TDMPP (90 MBq, 22 μCi) in wild-type rats 2, 4 and 24 h post injection

As shown in figure 7 the rats showed accumulation of the radiotracer in the skeletal system and specially the joints 2 h post injection. This was not observed in the distribution study since the bone sample is taken from the body of the thighbone and not the bone ends. Knee joints and the shoulders are significantly sites of uptake. Interestingly myocardial uptake is observed at this time point which is in agreement with the biodistribution study. However after 4 h most of the activity is washed out through the urinary tract and kidneys are the only obvious organs as well as the bladder. 24 h after injection the only accumulating site is the bladder and most of the activity is washed out from the body after 24 h. 

Our first impression of the SPECT image was that the knee uptake can be a consequence of release of free Ga-67 cation from the radiolabeled compound as shown in free Ga- 67 data the uptake can be more than 2% after 24 h, however, the biodistribution data from the radiotracer accumulation shows that bone uptake is lower than 0.3% at all time intervals, thus the real joint uptake mechanisms is different for the free Ga-67 uptake. 

Previous investigations on the porphyrin tissue uptake using *ex-vivo *fluorescence measurements has demonstrated the predominant accumulation of porphyrins in the synovium as well as cartilage tissues taken from knee joints. However, the fluorescence spectra features indicated that the composition of porphyrins detected in the cartilage tissues was different than that in the synovial tissues (24). 

The expected tracer uptake in tumor models is not a consequence of cellular turn-over, because the tracer does not target protein/DNA synthesis. The proposed mechanism of porphyrin uptake in the tumor cells is explained through the LDL receptors as reported earlier (25). 

## Conclusion

Total labeling and formulation of [67Ga]- TDMPP took about 60 min (RCP >99% ITLC, >99% HPLC, specific activity: 12-15 GBq/mmol). The complex was stable in final formulation and human serum at least for 24 h. At the pH.7, the log P was 1.63. The biodistribution of the labeled compound in vital organs of wild-type rats was studied using scarification studies and SPECT imaging up to 24 h. A detailed comparative pharmacokinetic study performed for ^67^Ga cation and [^67^Ga]- TDMPP. The complex is mostly washed out from the circulation through kidneys. Myocardial uptake was significantly observed by SPECT and biodistribution studies. Knee and shoulder joints demonstrated significant activity uptake in 2 h post injection. Higher water solubility of the complex due to ionic nature of the complex is an advantage for rapid wash-out of the complex from the system. 
